# Case Report: Polymyxin B-induced anaphylactic shock

**DOI:** 10.3389/fphar.2025.1594127

**Published:** 2025-06-25

**Authors:** Weiqian Liu, Bo Gao, Shaowei Xie, Yu Yin

**Affiliations:** ^1^ Department of Rehabilitation Medicine, Hebei General Hospital, Shijiazhuang, Hebei, China; ^2^ Hebei Provincial Key Laboratory of Cerebral Networks and Cognitive Disorders, Hebei General Hospital, Shijiazhuang, Hebei, China; ^3^ Department of Urinary Surgery, Hebei General Hospital, Shijiazhuang, Hebei, China

**Keywords:** case report, polymyxin B, anaphylactic shock, adverse drug reactions, antibiotics

## Abstract

**Rationale:**

Polymyxin B is a widely used antibiotic in clinical practice, and anaphylactic shock represents a severe adverse drug reaction. Reports of polymyxin B-induced anaphylactic shock are rare in the existing literature.

**Patient concerns:**

A 67-year-old male patient in the recovery phase of cerebral infarction presented with fever. The suspected pathogen responsible for his pulmonary infection was drug-resistant *Pseudomonas aeruginosa*, which is resistant to polymyxins. Following the administration of polymyxin B, the patient experienced anaphylactic shock.

**Diagnosis:**

Consider the anaphylactic shock induced by polymyxin B.

**Interventions:**

Adrenaline was administered via intramuscular injection, and a micro-pump of metaraminol was utilized for infusion. Fluid resuscitation, intravenous dexamethasone administration, and nebulized budesonide were also performed. Consequently, the patient’s blood pressure gradually returned to normal levels.

**Outcome:**

The patient was transferred to the respiratory department for further treatment.

**Lessons:**

To prevent serious adverse reactions, it is essential to strictly adhere to indications and contraindications to avoid misuse and minimize the occurrence of adverse effects. Prior to medication administration, a thorough inquiry into the patient’s drug allergy history and family history should be conducted.

## 1 Introduction

Polymyxin B is a classic antibacterial agent that can be used to treat infections caused by Gram-negative bacilli, such as *Acinetobacter* baumannii and *Pseudomonas aeruginosa* (Guangyu et al., 2015). There are five types of polymyxins: A, B, C, D, and E. Currently, only polymyxin B and polymyxin E have been approved for clinical use; among these, the antibacterial activity of polymyxin B is significantly superior to that of polymyxin E (Yongming and Ying, 2017; Xu, 2019). Sulfate polymyxin B is a hydrophilic molecule with a positive charge that possesses the ability to bind endotoxins. This binding inactivates endotoxins and inhibits their production, making it an important medication for combating infections (Wang et al., 2020). Common adverse reactions associated with sulfate polymyxin B include nephrotoxicity and neurotoxicity; less frequently observed effects may include fever, eosinophilia, and rash (Bing, 2015). Herein we report a case of allergic shock induced by polymyxin B.

## 2 Case presentation

The patient is a 67-year-old male who was admitted on 14 November 2024, due to impaired mobility of the right limbs for over 1 month. The diagnoses are as follows: 1. Hemiplegia; 2. Dysphagia; 3. Cerebral infarction in the recovery phase; 4. Coronary atherosclerotic heart disease with a history of coronary artery stent implantation; 5. Asthma; 6. Renal calculi. The patient has a known allergy to cephalosporins and “Dan Shen.” He also has a medical history of anaphylactic shock due to cephalosporin hypersensitivity.

On November 16, the patient presented with increased sputum production and fever, with a maximum body temperature reaching 38.3°C. Hematological analysis demonstrated a white blood cell count (WBC) of 9.76 × 10^9/L, neutrophils (NEUT#) at 5.75 × 10^9/L, lymphocytes (LYMPH#) at 2.88 × 10^9/L, and procalcitonin PCT (quantitative fluorescence method) <0.04 ng/mL.

The patient’s medical history indicates that they have been receiving piperacillin-tazobactam for the treatment of pneumonia at an external facility for over 10 days. Therefore, the antibiotic regimen has been escalated to meropenem. The patient presented with intermittent fever. Chest CT scan and three-dimensional reconstruction indicated a high likelihood of scattered inflammation in both lungs, along with partial bronchiectasis. There was thickening observed in the bilateral pleura and interlobar pleura. Multiple slightly enlarged lymph nodes were noted in the mediastinum, as well as a nodule in the anterior mediastinum, for which follow-up examination is recommended. Additionally, there was a small amount of pleural effusion on both sides and slight thickening of the esophageal wall. A high-density shadow was identified in the region of the coronary artery; clinical correlation is advised. The patient underwent sputum suction via electronic bronchoscopy and bronchoalveolar lavage through bronchoscopy. The culture of bronchoalveolar lavage fluid indicated the presence of *P. aeruginosa* resistant to carbapenem antibiotics, with a sensitivity to colistin (CS) at 2 μg/mL.

Therefore, on December 3rd, it was decided to administer nebulized colistin sulfate B (10 mL: 50 WU*1, Shanghai Pharmaceutical First Biochemical Pharmaceutical Co., Ltd.) at a dosage of 500,000 units every 12 h for anti-infective treatment. However, the patient continued to experience excessive sputum production, airway spasms, and significant wheezing, with sputum being difficult to expectorate. On December 5th, the treatment was modified to intravenous colistin. Following the administration of colistin, a rash developed on the patient’s right forearm, accompanied by a subsequent drop in blood pressure to 59/33 mmHg. Immediate interventions included intramuscular injection of epinephrine, infusion of norepinephrine via a micro-pump, fluid resuscitation, intravenous dexamethasone administration, and nebulization with budesonide. The patient’s blood pressure gradually returned to normal levels. Consultation from the Department of Critical Care Medicine: The patient is suspected to be experiencing anaphylactic shock due to an allergy to polymyxin. Currently, under high-dose norepinephrine infusion, blood pressure has risen to 111/75 mmHg. The patient’s skin appears dry throughout, and there is no edema in the extremities. An arterial blood gas analysis was performed, and appropriate fluid resuscitation was administered. The dosage of norepinephrine will be gradually reduced while closely monitoring changes in the patient’s blood pressure and heart rate. Subsequently, the patient was transferred to the Respiratory Department for further treatment.

## 3 Discussion

Polymyxin B is a peptide antibiotic discovered in 1947. However, due to its severe nephrotoxicity, it has gradually been replaced by other newer antimicrobial agents. In recent years, the increase in infections caused by multidrug-resistant Gram-negative bacteria (Hu et al., 2018) has led to the emergence of new drugs, albeit limited and often expensive; consequently, polymyxin-class antibiotics have found renewed application in clinical settings (Chinese XDR Consensus Working Group et al., 2016). As supportive data for the use of polymyxin B in infected patients continues to accumulate, it has become a last-line treatment for hospital-acquired pneumonia caused by multidrug-resistant, extensively drug-resistant, or pan-drug-resistant pathogens (Exner et al., 2017).

According to the Naranjo Adverse Drug Reaction Probability Scale (Table 1), which assesses the likelihood of adverse reactions related to medications, the algorithm yielded a score of 9, indicating a definite association. With the increasing clinical application of polymyxin B, cases of adverse reactions have garnered growing attention. Ning et al. (2022) a case involving a patient with stage 5 chronic kidney disease (CKD) who experienced severe respiratory paralysis during treatment with polymyxin B; this occurred after the administration of 11 doses. Chin et al. (2023) another patient who developed hypercapnic respiratory failure leading to neuromuscular dysfunction following treatment for multidrug-resistant *Escherichia coli* bacteremia with polymyxin B. Additionally, Zheng et al. (2018) a case of excessive skin pigmentation induced by polymyxin B in a 21-year-old female patient (PMB-iSH).

**TABLE 1:** Naranjo ADR evaluation scale.

Naranjo ADR evaluation scale. Related questions			
Related questions	Score		
	Yes	No	Unknown
1. Is there any conclusive report before this ADR?	1√	0	0
2. Do the ADR occur after the use of suspect drugs?	2√	−1	0
3. Do the ADR relieve after drug withdrawal or use of antagonist?	1√	0	0
4. Does the ADR recur after reuse of the suspect drug?	2√	−1	0
5. Are there other reasons that can cause the ADR independently?	−1	2	0
6. Does the ADR repeat after the application of placebo?	−1	1	0
7. Does the drug reach toxic concentration in blood or other body fluids?	1	0	0
8. Is the ADR aggravated (relieved) with the increase (decrease) of dose?	1√	0	0
9. Has the patient ever been exposed to the same or similar drugs and had similar reactions	1√	0	0
10. Is there any objective evidence to confirm the reaction?	1√	0	0
Total score	9		

Note: The total score ≥9 shows that the causal relationship of adverse drug reactions is definite; the total score 5–8 is probably or likely to be relevant; the total score 1–4 is possible to be relevant; the total score ≤0 is doubtful to be relevant.

ADR, adverse drug reactions.

This patient experienced bronchospasm during nebulization with polymyxin, which is considered a drug-related adverse reaction (Table 2). Previous studies suggest that the nature of bronchospasm occurring during the nebulization of polymyxin is associated with multiple mechanisms. These factors include direct chemical irritation, histamine release, airway hypersensitivity, stimulation from chemicals or foam generated during the nebulization process, and hypertonicity in the airways (Alothman et al., 2005). Nebulized inhalation of polymyxin can induce bronchospasm even in patients without asthma or related medical history; however, if such conditions are present, the risk may be significantly increased (Alothman et al., 2005). Management of bronchospasm typically necessitates discontinuation of the medication, administration of bronchodilators, and supplemental oxygen. Some clinical trial reports indicate that the incidence of bronchospasm among patients receiving polymyxin is between 1% and 5%. However, these figures may vary depending on the patients' underlying health conditions, such as chronic obstructive pulmonary disease (COPD) or asthma (Wang et al., 2022). The incidence of allergic reactions is approximately 3%–10%. However, some studies indicate that in certain circumstances, the incidence may rise to as high as 15%, particularly when patients have a known history of drug allergies (Ding et al., 2024).

**TABLE 2:** Clinical course timeline.

Date	Clinical Event/Observation	Diagnostic/Intervention summary
November 16	Productive cough and fever (up to 38.3°C)	blood count, PCT; continued anti-infective therapy
Nov 17–30	Poor response after >10 days of antibiotics	Piperacillin-tazobactam had been used at an outside hospital
December 1	Persistent symptoms	Antibiotics escalated to meropenem
December 2	Chest CT showed diffuse inflammation and bronchiectasis	Chest CT + 3D reconstruction
December 3	Underwent bronchoscopy with sputum suction and BAL	BAL culture: carbapenem-resistant *P. aeruginosa* (colistin sensitive); initiated nebulized colistin B
December 4	Profuse sputum, airway spasm, poor expectoration	Planned switch to intravenous colistin
December 5	Allergic reaction after IV colistin	Rash on right forearm, BP dropped to 59/33 mmHg; emergency management (IM epinephrine, vasopressors, fluid resuscitation, IV dexamethasone, nebulized budesonide); diagnosed as anaphylactic shock due to colistin allergy

Allergic shock is a severe, life-threatening systemic allergic reaction that typically occurs rapidly following exposure to an allergen. The mechanism by which polymyxin B induces allergic shock may be related to the drug itself or its impurities acting as haptens, which bind with proteins in the body to form complete antigens. This process triggers the production of specific IgE antibodies. Upon re-exposure to polymyxin B, these antigens interact with IgE antibodies on the surface of mast cells and basophils, leading to the release of bioactive substances such as histamine and leukotrienes from these cells. This cascade results in a series of pathophysiological changes including vasodilation, increased capillary permeability, and smooth muscle contraction, ultimately culminating in allergic shock (Falagas and Kasiakou, 2006).

From a preventive perspective, it is crucial to thoroughly inquire about the patient’s allergy history. Case reports and small observational studies have described rare instances of colistin-induced hypersensitivity reactions, including rash, bronchospasm, and hypotension following intravenous or aerosolized administration. However, no large-scale cohort or pharmacovigilance studies have yet provided a clear incidence rate of these reactions.

For instance, in this case, the patient has a prior history of anaphylactic shock due to cephalosporin allergy, necessitating greater caution in subsequent medication administration.

## 4 Conclusion

Although polymyxin B demonstrates certain advantages in treating pulmonary infections, the serious adverse reaction of anaphylactic shock cannot be overlooked. Clinicians must fully understand its associated risks during use, strictly adhere to indications for treatment, and prepare adequately for prevention and emergency response measures to ensure the safety of medication administration for patients.

## Data Availability

The original contributions presented in the study are included in the article/supplementary material, further inquiries can be directed to the corresponding author.

## References

[B1] Alothman G. A. Ho B. Alsaadi M. M. Ho S. L. O'Drowsky L. Louca E. (2005). Bronchial constriction and inhaled colistin in cystic fibrosis. Chest 127, 522–529. 10.1378/chest.127.2.522 15705991

[B2] Bing Lü (2015). Clinical analysis of 47 cases of ventilator-associated pneumonia. Chin. J. Health Stand. Manag. 6 (27), 134–135.

[B3] Chin A. X. Y. Ng K. W. P. Chan Y. C. Goh Y. Rathakrishnan R. (2023). Polymyxin-induced neuromuscular weakness: a case report. Front. Neurol. 15, 1342419. 10.3389/fneur.2024.1342419 PMC11004478 38601335

[B4] Chinese XDR Consensus Working Group, Guan X. He L. Hu B. Hu J. Huang X. (2016). Laboratory diagnosis, clinical management and infection control of the infections caused by extensively drug-resistant Gram-negative bacilli: a Chinese consensus statement. Clin. Microbiol. Infect. 22, S15–S25. 10.1016/j.cmi.2015.11.004 26627340

[B5] Ding P. Li H. Nan Y. Liu C. Wang G. Cai H. (2024). Outcome of intravenous and inhaled polymyxin B treatment in patients with multidrug-resistant gram-negative bacterial pneumonia. Int. J. Antimicrob. Agents 64 (4), 107293. 10.1016/j.ijantimicag.2024.107293 39094752

[B6] Exner M. Bhattacharya S. Christiansen B. Gebel J. Goroncy-Bermes P. Hartemann P. (2017). Antibiotic re sistance: what is so special about multidrug-resistant gram-negative bacteria. GMS Hyg. Infect. Control 12, Doc05. 10.3205/dgkh000290 28451516 PMC5388835

[B7] Falagas M. E. Kasiakou S. K. (2006). Toxicity of polymyxins: a systematic review of the evidence from old and recent studies. Crit. CARE 10 (1), R27. 10.1186/cc3995 16507149 PMC1550802

[B8] Guangyu L Wenfang J. Tianbin C. Youhua Z. (2015). Clinical observation of amikacin pulmonary lavage via fiberoptic bronchoscopy in the treatment of ventilator-associated pneumonia caused by multidrug-resistant Acinetobacter baumannii. Chin. Med. Guide 13 (22), 38–40. 10.15912/j.cnki.gocm.2015.22.025

[B9] Hu F. P. Guo Y. Zhu D. M. Wang F. Jiang X. F. Xu Y. C. (2018). Antimicrobial resistance profile of clinical isolates in hospitals across China: report from the CHINET Surveillance Program. Chin. J. Infect. Chemother. 18 (3), 241–251. 10.16718/j.1009-7708.2018.03.001

[B10] Ning Y. Chu Y. Wu Y. Huang Y. Wang C. Jiang L. (2022). Case Report: respiratory paralysis associated with polymyxin B therapy. Front. Pharmacol. 13, 963140. 10.3389/fphar.2022.963140 36105193 PMC9465242

[B11] Wang P. Liu D. Sun T. Zhang X. Yang J. (2022). Pharmacokinetics and pharmacodynamics of polymyxin B and proposed dosing regimens in elderly patients with multi-drug-resistant Gram-negative bacterial infections. Int. J. Antimicrob. Agents 60 (5-6), 106693. 10.1016/j.ijantimicag.2022.106693 36375775

[B12] Wang W. Ding W. Huang J. Chen L. Yu F. (2020). Observation of the effects of meropenem combined with polymyxin B sulfate on patients with pan-drug-resistant Acinetobacter baumannii bacteremia and its impact on PCT and CRP. General Pract. Med. Clin. Educ. 18 (3), 212–214. 10.13558/j.cnki.issn1672-3686.2020.003.006

[B13] Xu L. (2019). Evaluation of the clinical efficacy and safety of combined sulbactam polymyxin B and meropenem in patients with pan-resistant acinetobacter baumannii bacteremia. Anti-Infection Pharmacol. 16 (10), 1743–1745. 10.13493/j.issn.1672-7878.2019.10-025

[B14] Yongming F. Ying L. (2017). Clinical efficacy analysis of amikacin combined with cefoperazone-sulbactam sodium in elderly patients with ventilator-associated pneumonia. Anti-infective. Pharmacology 14 (1), 155–157. 10.13493/j.issn.1672-7878.2017.01-056

[B15] Zheng G. Cao L. Che Z. Mao E. Chen E. He J. (2018). Polymyxin B-induced skin hyperpigmentation: a rare case report and literature review. BMC Pharmacol. Toxicol. 19, 41. 10.1186/s40360-018-0226-1 29973293 PMC6032769

